# The STARTEC Decision Support Tool for Better Tradeoffs between Food Safety, Quality, Nutrition, and Costs in Production of Advanced Ready-to-Eat Foods

**DOI:** 10.1155/2017/6353510

**Published:** 2017-12-04

**Authors:** Taran Skjerdal, Andras Gefferth, Miroslav Spajic, Edurne Gaston Estanga, Alessandra de Cecare, Silvia Vitali, Frederique Pasquali, Federica Bovo, Gerardo Manfreda, Rocco Mancusi, Marcello Trevisiani, Girum Tadesse Tessema, Tone Fagereng, Lena Haugland Moen, Lars Lyshaug, Anastasios Koidis, Gonzalo Delgado-Pando, Alexandros Ch. Stratakos, Marco Boeri, Cecilie From, Hyat Syed, Mirko Muccioli, Roberto Mulazzani, Catherine Halbert

**Affiliations:** ^1^Norwegian Veterinary Institute, Oslo, Norway; ^2^IRIS, Barcelona, Spain; ^3^University of Bologna, Bologna, Italy; ^4^Queens University, Belfast, UK; ^5^Matbørsen, Stokke, Norway; ^6^Kohinoor, Dublin 24, Ireland; ^7^Forno Romagnolo, Poggio Torriana, Italy; ^8^Halbert Research, Ireland

## Abstract

A prototype decision support IT-tool for the food industry was developed in the STARTEC project. Typical processes and decision steps were mapped using real life production scenarios of participating food companies manufacturing complex ready-to-eat foods. Companies looked for a more integrated approach when making food safety decisions that would align with existing HACCP systems. The tool was designed with shelf life assessments and data on safety, quality, and costs, using a pasta salad meal as a case product. The process flow chart was used as starting point, with simulation options at each process step. Key parameters like pH, water activity, costs of ingredients and salaries, and default models for calculations of* Listeria monocytogenes*, quality scores, and vitamin C, were placed in an interactive database. Customization of the models and settings was possible on the user-interface. The simulation module outputs were provided as detailed curves or categorized as “good”; “sufficient”; or “corrective action needed” based on threshold limit values set by the user. Possible corrective actions were suggested by the system. The tool was tested and approved by end-users based on selected ready-to-eat food products. Compared to other decision support tools, the STARTEC-tool is product-specific and multidisciplinary and includes interpretation and targeted recommendations for end-users.

## 1. Introduction

Convenient foods today include prepared full meals and ready-to-eat products like deli salads, wraps, baguettes, and sushi, all containing several ingredients. The consumption of such products has increased [1, 2], and they are expected to be available where people buy other foods; they should preferably have a long shelf life in addition to desired sensory quality, acceptable price, and guaranteed food safety. The change from traditionally prepared foods towards full meal convenience and ready-to-eat products has the consequence that ingredients are processed and stored under conditions where they are likely to spoil at different rate and/or pathogenic bacteria have other opportunities to grow than in the single ingredients [3–5]. Food business operators have to deal with these challenges and make many decisions regarding suppliers, ingredients, food formulations, process conditions, distributors, and customers to optimise quality, safety, and cost. HACCP and internal control routines such as GMP and GHP are used, but even under “business as usual conditions” this is challenging, as many ingredients and processing operations are needed to produce deli salads, convenience meals, and other RTE products. Decisions are even more challenging in ad hoc situations, for instance, when the preferred suppliers and ingredients are not available, if unintended deviations in process or storage conditions occur, and/or when customers demand a higher safety and quality level than the standard.

Regarding food safety of ready-to-eat foods, the European food authorities are especially concerned about the bacterium* Listeria monocytogenes* that causes the disease listeriosis. The mortality rate of listeriosis is high and the symptoms are severe, in particular for unborn babies, the elderly, the immunocompromised patients, and consumers with underlying diseases [6–9]. A general increase of listeriosis cases has been observed during the last decades [7] and several outbreaks of listeriosis related to ready-to-eat foods, some of them at hospitals, have taken place [7, 10, 11]. Criteria for* L*.* monocytogenes* in ready-to-eat foods are now included in the European Food Law, regulation 2073/2005 [12]. These criteria take into account the fact that* L*.* monocytogenes* can grow in many products under cold storage conditions. The maximum level in ready-to-eat products is 100 cfu/g in products which do not support growth and 100 cfu/g at the last day of shelf life under reasonably foreseeable storage conditions in products that do support growth, with absence in products intended for medical use. The food providers need to assess their products in terms of* Listeria* growth potential or growth rate under reasonably foreseeable conditions as well as to document that the* Listeria* counts are within the legal limits. Guidelines for this purpose have been developed and implemented in EU [13, 14]. This topic is in itself challenging, but it is only one of many parameters to consider for food business operators.

Several decision support tools with predictive models for food pathogens have been developed during the last decades, like ComBase, Sym'Previus, FDA-iRisk, FSSP, MicroHibro, PMM-Lab, Danish meat model, GinaFit, and others [15–22]. The tools, recent developments and models, and benefits and barriers for use are described in extensive reviews [23–26]. These tools can be used to estimate the growth and survival of* L. monocytogenes* and other pathogens in foods. Despite the high quality and availability of these tools, the number of users in industry is low [24]. Some tools are very advanced and developed for risk assessment on an overall level [24, 26], while the need and duty of food producers are to make sure that their products are in compliance with the legal limits, which means a HACCP and good hygienic practice approach rather than a detailed risk assessment [27, 28]. It is therefore likely that a tool similar to an expert system, which according to Filter et al. [23] consists of a knowledge base, inference engine with signal creation, and a user-interface with recommendations, would be more useful for food producers than a system that provides assessments but not consequences and recommendations. A review of Egan et al. [27] about impact of foody hygiene training supports this assumption. It describes that knowledge does not necessarily lead to changes in attitudes, behavior, and work practices. On this background, a possible reason for limited use or understanding of the existing decision support tools may be that most of them focus mainly on food safety and assessments and do not consider other important aspects of food manufacturing such as quality change and production costs.

The objective of the STARTEC project was therefore to develop tools, which means a prototype IT- (information technology-) tool and guidelines including possible corrective actions for use in food industry where several important factors need to be considered in parallel: food safety, quality, nutrition, and cost. In order to investigate how decisions are made today and which issues decision support is needed for, a mapping of industry processes was carried out in three companies of advanced ready-to-eat foods. Based on the observations, an IT prototype tool was designed and tested using full meal pasta salads as case studies. Finally, the tool was presented to experts and possible end-users to assess the relevance of the tool for industry. The methodology and outcome are presented in this paper.

## 2. Materials and Methods

### 2.1. Mapping of Decision-Making and Needs by Food Producers

**Figure 1:**
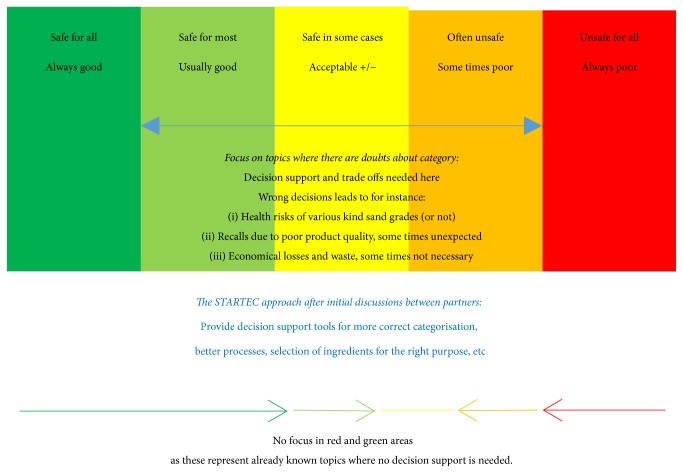
The initial approach of the STARTEC decision support tool and guidelines.

In order to develop a tool that was as relevant as possible for the intended end-users, a mapping of industry conditions was carried out to establish the critical food safety and quality challenges that small and medium sized enterprise (SME) deal with. The first step was a discussion of the context of the decision support tool (Figure 1) at a workshop where all the food producers and researchers in the team participated. During the next six months, researchers visited the companies for “walk and talk” meetings in the production facilities in order to observe the food hygiene challenges in commercial food production, as well as to investigate which kinds of decisions the industry needed assistance to do, which realistic corrective actions could be done, and how decisions were made in each of the companies. The researchers who carried out the walk and talk visits had competence in food hygiene. Together with the quality managers and leading staff in the companies they collected flow charts of the products the companies had selected as especially relevant and discussed the critical control points, likely deviations, and possible corrective actions in case of deviations. To obtain an overview of strategic thinking of quality, nutrition, and cost aspects, an interview guide was developed, using Lasagna production as a model product, as all companies produced Lasagna (Algorithm 1). The interview guide was applied in a semistructured way, as the purpose was to learn from the day-to-day food production activities across different companies.

### 2.2. Data of Models for Pasta Salad for Implementation in the Tool

Based on the results from the industry mapping, experiments for food safety, quality, and cost parameters were designed using deli salads as case product. Two full meal pasta salads, consisting of cooked pasta, meat, vegetables, cheese, and a sauce of oil, lemon, salt, and pepper in two different formulations were applied. The salads were prepared in the lab from ingredients provided either from the companies or from retail, packed in MAP (CO_2_/N_2_) or air according to the practices in the companies and stored at 4 or 12°C. For validation, salads produced and packed in the companies were inoculated through a septum and stored at three different temperatures.

#### 2.2.1. Food Safety Parameters, Models, and Technologies for Corrective Actions

Among the food safety parameters,* L. monocytogenes *was focused particularly [29–33]. Challenge studies were carried out based on the European Guidelines [14] in order to investigate the growth rate and growth potential of the bacterium in the model product. The pH, water activity, content of lactic acid bacteria, and total viable counts were measured and used for development and primary and secondary models for growth were developed, as described by De Cesare et al. [29]. The approach was to develop a model as simple and with as few parameters as possible, because this means greater parsimony and thus greater explanatory power. In the case that they are devoted to describe the population growth or, alternatively, the variation of dynamics parameters according to environmental conditions change, microbial models have been broadly categorized into primary and secondary models [34].

Fifteen datasets obtained by challenge experiments in different scenarios, according to the STARTEC-tool approach, were analyzed to select and calibrate primary and secondary models suitable to describe the food matrix. Different primary growth models were tested to fit the experimental data for* L. monocytogenes* growth, including the Baranyi model, with or without lag and stationary phases [35, 36], Rosso model [37], and a simple exponential growth model. The secondary models describe the dependence of primary model parameters on environmental conditions. The environmental conditions investigated in this study were storage temperature and product pH. Among secondary models, the square root model allows predicting the *μ*_max_ values at different temperatures [38]. A more complex secondary model is the gamma model of Rosso et al. [37], in which the concept of optimum growth rate (*μ*_opt_) is defined and the dependence over temperature and pH includes optimum and maximum values of the growth parameters. Goodness of fit was assessed considering the coefficient of determination (*R*^2^) and the root mean square error (RMSE) between the observed data and the values predicted by the model.

Some studies of* Bacillus, Salmonella, Staphylococcus,* and toxins in deli salads were carried out in order to develop the tool for more food safety hazards [31, 39, 40]. Models were not implemented in the tool, but some studies were included as guidelines for corrective actions. For the same purpose, novel volumetric and surface active preservation technologies on RTE foods or ingredients were investigated. The application of these technologies showed promising results on the improvement of the microbiological safety of RTE products or ingredients without compromising the quality and sensory characteristics. High Pressure Processing (HPP), novel Continuous Microwave Heating System, and biopreservation with* W. viridescens* were investigated along with antimicrobial packaging by coriander oil and Cold Plasma (CP) [32, 33, 41, 42].

#### 2.2.2. Quality Changes of Deli Salads

The sensory changes of the pasta salads were assessed using a quality index form. Laboratory staff (4–6 persons) scored individually the salads throughout the storage period on the attributes freshness, colour, odour, and texture. The scales were for all attributes (1): inedible-unacceptable, very poor, and strong defects, (2): poor, major defects; (3): fair, acceptable defects, (4): good, acceptable defects, and (5): typical attribute, very good, and without defects. Each assessor scored also their own overall acceptability on a scale from 1 (dislike extremely) to 9 (like extremely). The studies were done in three labs in three countries. Data from more than 30 datasets were collected and the reduction in quality score for each storage scenario was fitted to a quadratic model and inserted in the STARTEC-tool. Limit values for good, sufficient, and unacceptable were estimated based on obtained scores.

#### 2.2.3. Nutrition Data

The contents of fat, carbohydrate, protein, and micronutrients of the ingredients of pasta salads were obtained from UK and USA public data collections [5, 43]. Salad nutritional composition was calculated from the ingredients, and total energy was calculated from multiplying each total macronutrient content by their caloric content, that is, protein and carbohydrate 4 kcal/g whereas fat was 9 kcal/g.

Changes in macronutrient composition during storage under different conditions (time and temperature) are meaningless; thus our investigation was focused on micronutrient degradation. Vitamin C (L-ascorbic acid) is an important water soluble vitamin which plays an important role in the normal body function. It is an important antioxidant and is also enzyme cofactor for different biochemical reactions; intakes of vitamin C have been positively associated with iron status in older people [44]. Adequate vitamin C intake is essential in a healthy diet, as micronutrient needed for the normal body function but also from the point of view of disease prevention. Furthermore, vitamin C is more sensitive than other micronutrients. Therefore, it can be assumed that if vitamin C retention is high, other vitamins will have similar or higher retention percentage [45]. A model based on the degradation kinetics of ascorbic acid was obtained after research on RTE pasta salads under different processing and storage conditions. Arrhenius equation was employed when degradation was temperature dependent. All these data were gathered and inserted in the IT-tool [46].

#### 2.2.4. Production Costs and Willingness to Pay

The production costs for deli salads, distributed on the categories ingredients, energy, salary, packaging, and distribution were collected from the companies and the total costs calculated. A separate study to address the willingness to pay was carried out in a consumer survey in six countries, using different attributes of meat ingredients in Lasagna as case. The study is described by Agnoli et al. [47]. The amount of money the consumers was willing to pay for their preferred alternative compared to the standard alternative in this study was used to set limits for acceptable and unacceptable costs for deli salads.

### 2.3. Development and Testing of the IT-Tool

An IT-tool was developed to a prototype level, based on inputs from the industry mapping, generation of experimental data, and model developments. Python codes were applied. The database of the tool consisted of parameters, units, ingredients, treatments, formulas (models), aggregated formula, experimental data, and flow charts. A module for drawing flow charts based on ingredients and treatments in the database was included. In the simulation module, formulas can be selected and customized for each treatment; however, this has been fully implemented for mixed deli salad, only. A user-interface which provides an overview of the product characterisation, customization of models, and settings and limit values for categorization of simulation outputs in a traffic light system, corresponding to green-good, yellow-sufficient, and red-not acceptable/corrective action needed, was designed. All parameters can be simulated and the outputs can be given as either curves or a summary. The different modules are presented in more detail in the Results and Discussion.

The user-friendliness and relevance of the tool was tested as follows; a restricted version of the tool, tutorials, and a set of exercises were prepared and sent to 10 internal partners and experts and 39 external companies who had all showed interest in the tool during conferences. The test persons were asked to fill a web based questionnaire after the exercises.

## 3. Results and Discussion

### 3.1. Results of Industry Mapping: Scope of the STARTEC-Tool

The initial idea of the STARTEC-tool concept (Figure 1) was to categorize the food safety, quality, and costs of the sum of ingredients, processes, and storage conditions in good, sufficient, and not acceptable/corrective action needed. The industry partners in the team found the approach useful. They pointed out that the tool needed to focus on real challenges to be useful and, further, that decision support was needed on topics where there was doubt of the categorization. The essential step in the tool development was to categorize products correctly in red, yellow, and green area, corresponding to unacceptable, sufficient, and good for various parameters describing food safety, quality, and costs. The categories should preferably be possible to adapt to intended consumer groups, including vulnerable consumers and customers demanding a long shelf life and a higher quality or safety standard. Further, it was desired that the tool should provide corrective actions to move from red and yellow to green category. It was pointed out that the corrective actions should not be theoretical ones but possible for the industry to apply. A longer cooking period to improve food safety was given as an example of a not realistic corrective action for products that would get a too soft or dry texture, that is, a lower quality category, if heated longer. A stricter sampling regime, a mild heat treatment process for the critical ingredient, and to formulate a product without the critical ingredient for food safety and advice to the customer to add the ingredient shortly before serving were all considered as useful corrective actions [31]. It was pointed out that the options for ad hoc changes in product formulations and processes would not be acceptable, as the products specifications that agreed with customers were strict and ad hoc changes would lead to confusion and likelihood of mistakes. Therefore, the most relevant use of a tool would be for strategic decisions, product development, and assessment of* unintended* deviations.

The “walk and talk visits” in the production facilities showed, as expected, an extreme complexity in production of mixed ready-to-eat and ready-to-heat product, as many different products with branched flow schemes had to be produced within a limited timeframe and space, following strict specifications from the customers. In this complexity, the approach taken by the food producers was to reduce the likelihood of deviations as much as possible and to have as large utilization of the production capacity and facility as possible, without compromising food quality or safety. Standardized production of many products was done to adapt to the different customer demands rather than to adapt a product on an ad hoc basis. Decisions were made by few persons who were especially trained according to HACCP and other food safety and processing management systems. To increase the production capacity, precooked and cut ingredients provided by suppliers were sometimes used. Such ingredients lowered the likelihood of mistakes in product composition and if frozen, the ingredients accelerated the cooling process, which was a benefit. From a food hygiene point of view, on the other hand, they represented a potential food safety risk as contamination originating from the supply chain would not be eliminated during production even if the other parts of the products were sufficiently heat treated. The companies who used precooked and frozen ingredients were aware of this and had appropriate HACCP systems to deal with the challenge.

From a food hygiene point of view, the main challenge for producers of complex ready-to-eat foods is the branched material and process flows and the links between products due to the fact that some ingredients and processes are used in different products, sometimes after a storage period internally. The observations in the walk and talk visits indicated that contamination at several steps in the production flow may occur, which means that critical control points were needed not only for heat treatment but also for the material handling, storage, and transport between each of the process steps. Thus, a decision support tool should consider a realistic sequence of process steps, and each branch in the flow chart should be possible to assess separately. The main need for decision support was a system which could provide an overview of compliance with food safety, nutrition, costs, and quality criteria set by the legislation, customers, or internally, with a reasonable safety margin.

For strategic decisions the mapping activities indicated that decision support tools would be useful for food industry in management of multidisciplinary dilemmas like the following:

- The option to increase the production capacity by using preprocessed ingredients provided from subcontractors or others versus the need of in-house food safety control of the products.
- The need of different products within a category versus the increased risk of mistakes by differentiated production and inherent complexities.
- Rapid cooling versus the risk of recontamination, that is, the risk of overcooking in some parts and survival of microbes including pathogens in other parts of the food.

The three companies in the STARTEC team are all successful in their domains, have high hygienic standards, and are located in different countries with different climatic conditions and food culture. Thus, even though the mapping is based on only three companies, we assume the similarities in challenges and decision procedures identified are a good basis for development of a decision support tool that can be useful for industry.

### 3.2. Selection of Sample Product for Design of the IT-Tool

**Figure 2:**
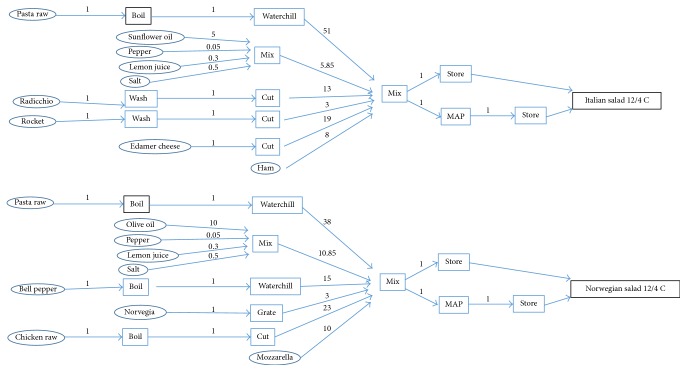
Flow charts of full meal pasta salads with Italian and Norwegian food producers. The oval rings describe ingredients, the squares treatments, and the numbers over the arrows the relative amount of ingredient in each process step.

Based on the outcome of the mapping activities, the IT-tool was designed with two formulations of mixed salads with pasta, meat, vegetables, and sauce as case products. These products and variations of them are widely produced in many countries and have all the characteristic challenges for mixed ready-to-eat products described above. The flow charts are shown in Figure 2. All ingredient groups are treated in different ways before mixing to the final product. The products can be differentiated by adjusting the amount ingredients or by replacing some of the ingredients. Use of preprocessed ingredients from a supplier instead of an in-house preprocessed one is common, according to our knowledge of the trade. Use of such ingredients will change the relevant branch of the flow chart, but the other branches remain. Commercial deli salads are packed with air or in CO_2_ enriched atmosphere and distributed under different temperature regimes. According to the guidelines for challenge studies for assessment of growth of* L. monocytogenes* in ready-to-eat products [14], the default test temperature in retail and by the consumer is 12°C while the producers normally recommend storage at 4°C. We therefore included packing in air and MAP and storage at 4°C and 12°C in the test scenarios.

### 3.3. STARTEC IT-Tool: Organization and Content

**Figure 3:**
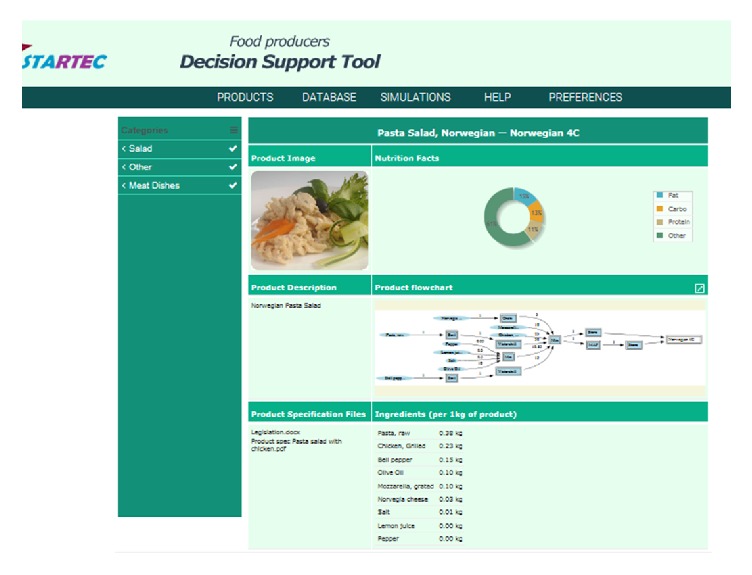
Front page of the STARTEC decision support tool after log-in.

The developed IT-tool is accessible on the Internet at http://startec-tool.iris.cat/startec/production with username and password. The front page after log-in consists of 7 panels, as shown in Figure 3. The products categories inserted in the tool are shown in the left panel. Clicking on the desired product fills the main part of the front page with relevant information for the product. The main part of the page gives an image of the product chosen, product descriptions, and a link to relevant documents, for example, legal documents, company internal control systems relevant for the product, product specifications, and text files of guidelines for improvement of food safety and quality. In the upper right panel, an overview of some nutrition facts of the product is given, based on information of the ingredients and recipe stored in the database. In the prototype version, the information is limited to protein, fat, carbohydrate, and energy, but the function can be extended with information on vitamins, trace minerals, and so forth. In the panel below, the process flow chart is given, and further below the list of ingredients and amounts of these are shown. The simulation module can be accessed from the flow chart in the right panel.

The tool consists of several modules that can be accessed from the tabs on the top of the user-interface. An introduction video to the tool is given in the “help” module, as well as on the link https://drive.google.com/file/d/0B9kCkwj1FAyOQ09pdWoxdGU2ZDg/view?pli=1. The database and simulations are the modules most developed. The interoperation of these modules makes the IT-tool more useful than the sum of these modules singularly.

#### 3.3.1. Database

**Figure 4:**
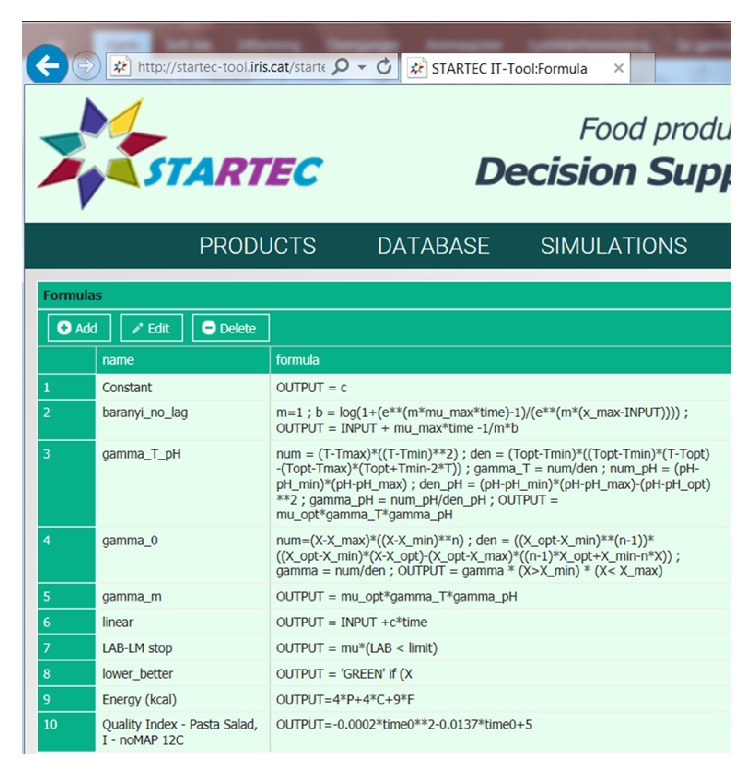
Examples of formulas inserted in the database.

The database consists of submodules. The* Product database module* stores the information that is relevant to the products the company offers, such as formulation, specification sheets, legislations, and photos. This is the source to the information on the front page. The* Measurement database module* allows storing data measured at different points along the preparation process of the products. The functionality of this module goes beyond that of a spreadsheet, as it allows (a) storing product flowcharts in a systematic way; (b) associating each measured aliquot with a specific point in the flowchart; (c) storing such information in a way which allows for systematic search with computers. Because of this, the database structure allows searches for measurement data with various criteria, and the result of such searches enables selecting data for modelling purposes or can serve as input to the simulation module. For instance, the composition overview on the front page and cost calculations in the simulation module (see below) are retrieved data from the database. The* Formula and Aggregated formula modules* store the models and algorithms used in the simulation. Some examples of formulas are shown in Figure 4. General models, like the baranyi_no_lag and the gamma_T_pH modules, are relevant for several products, and no product name is specified. The quality index, on the other hand, is strongly related to product, and this is indicated in the name of the formula. The users can access this module and add, edit, or delete a formula. This means that the user can apply the default models or enter other models to use for simulations.

#### 3.3.2. Simulation and Model Management Options

The* simulation module* can be considered as the engine of the tool, since this gives novel information to the users. The predictions and decision-making subsystem of the IT-Tool follow the general open philosophy, by not enforcing any particular algorithm to use. The tool provides the framework which may be filled with modelling and decision support logic. Therefore, the formulas for calculation of microbiological growth and other parameters are stored in the database and are not written in the source code. The source code only ensures that logic entered by users may be read from the database and executed in the system.

**Figure 5:**
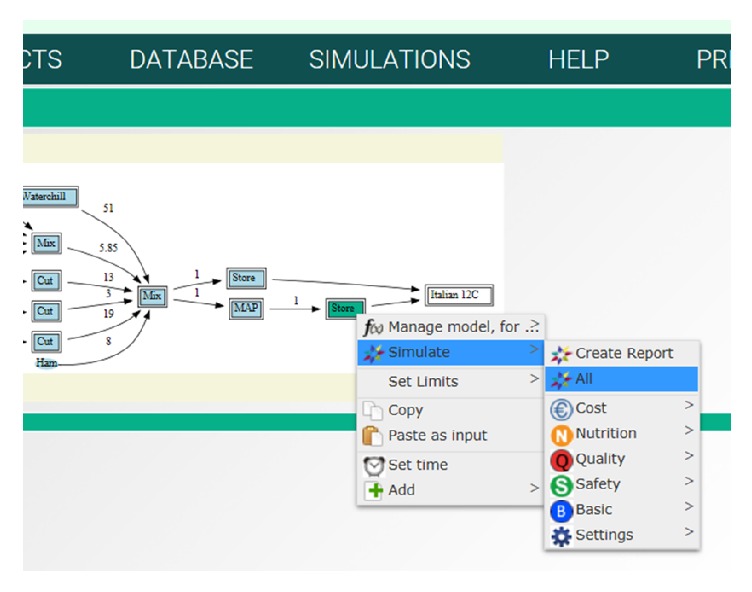
Procedure for simulation of parameters.

**Figure 6:**
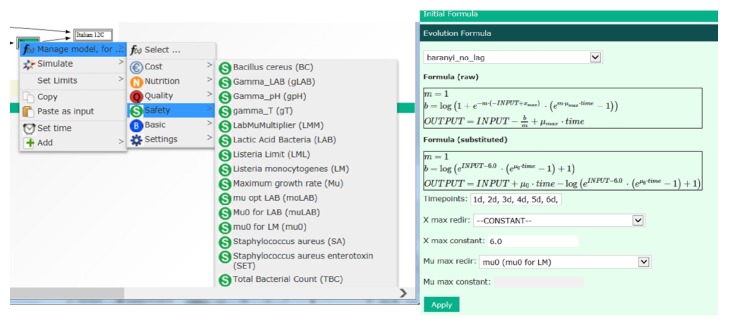
The procedure to manage the model of growth of* L. monocytogenes* from the simulation module.

**Figure 7:**
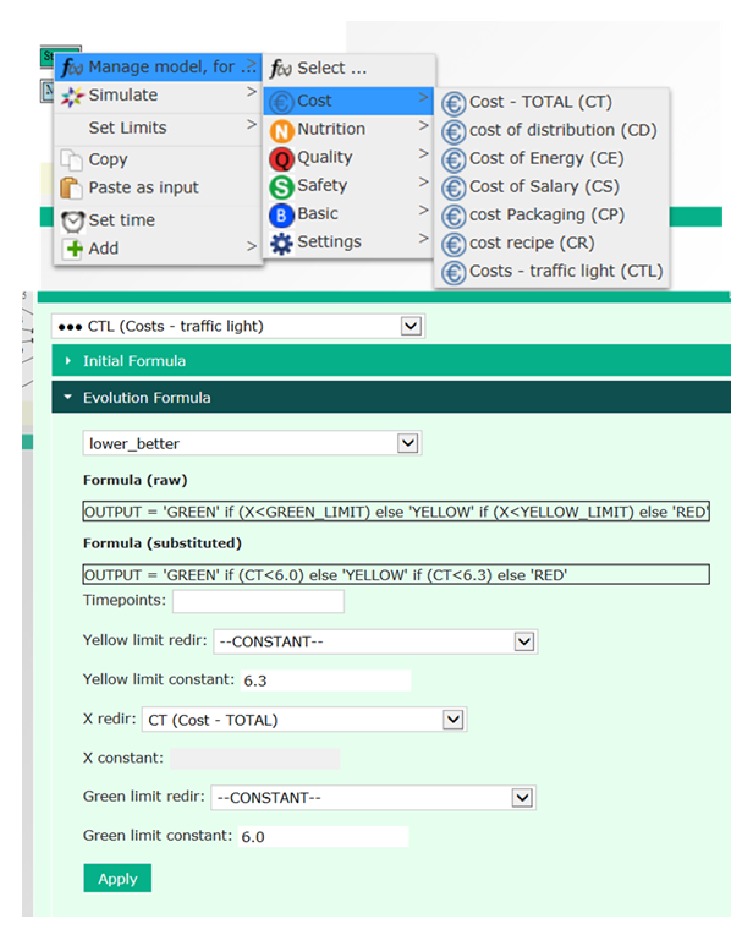
The cost parameters included in the tool. The threshold limit categorization for the traffic light system is indicated in the lower panel.

Simulations can be performed for single parameters, all parameters, or a category of parameters, as indicated in Figures 5, 6, and 7. The procedure is to click on the treatment box in the flow chart and select “simulate” from the drop-down menu and then “all” or a category of parameters. This procedure gives simulation outputs with default models and settings inserted in the tool. However, the models can be managed from the simulation module, as indicated for growth of* L. monocytogenes* in Figure 6. For this parameter, the Baranyi_no_lag was selected as default model as it fitted best the experimental data collected at 12°C [29]. The exponential model gave the best results for datasets collected at low temperature, but the Baranyi model was selected as default model in the STARTEC-tool also in this case since for very low values of *μ*_max_ it corresponded to the exponential model. The estimated *μ*_max_ values were included in the corresponding scenarios inside the STARTEC-tool as well as the dataset in order to show comparison between real data and implemented model. In the experimental studies, the measured *μ*_max_ values were compared with the predictions of secondary models proposed [29]. Despite that the gamma model was able to produce more accurate prediction according to the set of parameters, as temperature and pH, a simpler model as the square root secondary model was considered sufficiently accurate to give predictions in untested scenarios due to high complexity of deli salads. As different models were suited, the option to simulate with different models with different complexities were implemented in the tool. To change the module for simulation, the user can select model from the drop-down menu in the “manage model” window, as shown in Figure 6. Here, another formula can be chosen from the menu instead of the Baranyi_no_lag, for instance, the gamma_T_pH and gamma_m (see Figure 4). In case a model requires input values for pH, water activity, or other parameters, these can be retrieved from the database. Finally, the number of timepoints and other settings can be managed from this menu. Simulations and formulas can be managed for all parameters in a similar way. The system is designed to perform simulations for all treatments in the flow chart, but, in the present version, formulas have been entered for the storage of air and MAP packed salads after mixing, only. Option for simulation at dynamic conditions has not been included so far.

The categorization of the simulation output was done using a threshold value algorithm of the simulation data. The user can set his own threshold values or apply the default values, as indicated for the cost category in Figure 7, which shows the formula for the Cost-Traffic Light (CTL) model. The total production costs are summarized and the category changes when the costs exceed limit values. These limit values can be customized by the user, which is important as the costs, and willingness to pay is influenced by many factors not included in the tool. The Quality Traffic Light (QTL) is based on changes in the quality score during storage. The category changes from green to yellow and red when the scores drop from 5 (maximum value) to 4 and 3, respectively. The limit values can be customized, and if other quality indicators, for instance, rancidification, growth of spoilage bacteria, or off-odour formation, are inserted in the tool, the categorization can be based on changes in these parameters. For food safety, the default traffic light system is based on the categories for ready-to-eat foods in the microbial criteria in EU regulation and guidelines [12, 14], provided that the initial concentration is 1 cfu/g. Growth up to 0.5 log cfu/g was considered as no significant growth and therefore “good”; between 0.5 and 2.0 log cfu/g as sufficient; and to higher concentrations as unacceptable [14]. If the initial count is below 1 cfu/g, or other limit values for categorization, this can be inserted in the tool under “set limits” and under “manage models,” and the time until change of category will change. The 2 log cfu/g increase limit for growth is used in other IT-tools, like the Food Safety and Spoilage Predictor tool [48]. Even though there are legal limits for* L. monocytogenes* counts, some customers set stricter limits, and some producers want to increase the safety margins for products sold to hospitals and vulnerable consumers or take out the “sufficient” for other consumer segments. The limit values for categorization can therefore be customized for food safety in the tool. Suggested categories for* L. monocytogenes* growth in products intended for normal and vulnerable consumers are shown in Table 1.

**Figure 8:**
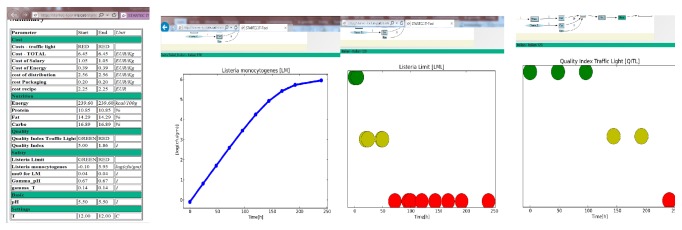
The simulation outputs in three versions, from left an overview report of all parameters selected, the simulation output curve for one parameter, and simulation outputs categorized in traffic light system to show compliance with criteria.

The tool provides simulation outputs in three formats, as shown in Figure 8. A summary report of all parameters gives the start and end values of each parameter and indicates if the category is green, yellow, or red. This output visualizes which parameters need to be improved in order to improve the category for the product as a whole. The other output is simulation curves for each parameter, and the third the traffic light for each parameter during storage. The summary report and combination of traffic light outputs for each category illustrate the multidisciplinary approach of the tool (Figure 8).

#### 3.3.3. Module for Background Information and Guidelines for Corrective Actions

Not all knowledge can be translated into models. A module of information about possible corrective actions and other guidelines based on research studies within the project was therefore implemented in the tool as short text documents designed for users in industry. The topics were chosen by industry partners. Some examples of the topics included are given below. Only the first sentences in the guidelines are given here, to illustrate the degree of detail and topics. Each guideline was linked to a text document of 2–5 pages with references for further information [31]. The documents have no legal authority, but they are included here to illustrate how background information for customization of parameters in the tool and possible corrective actions can be communicated to the user.

- There are multidisciplinary dilemmas and challenges associated with various process steps used in food production. Food safety risks can be reduced without compromising product quality and production costs if the complexity in production of advanced ready-to-(h)eat products and multidisciplinary dilemmas are taken into account. Challenges with branched process flow and use of preprocessed ingredients need to be included in the HACCP to ensure food safety. The likelihood of mistakes leading to risk of foodborne illness can be reduced by adapting product formulations, process steps, and staff training.
- The Performance Objective (PO) is a risk management concept we should become familiar with in the near future. The PO calculated in the STARTEC project for* Bacillus cereus* in spelt to be added in a mixed spelt salad packaged under modified atmosphere (MAP) corresponds to 1 cfu/g. This concentration level decreases to 2 cfu/10 g if the spelt is added to salads packaged under air. In cheese the POs calculated when this ingredient is added in a spelt salad packaged under air or MAP are 6 cfu/kg and 4 cfu/100 g, respectively.
- Food products and conditions can be categorized into red, yellow, and green, corresponding to unacceptable, marginal/sufficient, and good. Yellow and red categories indicate that a corrective action or special care has to be taken. Food safety categories can be set up based on the growth potential* of L. monocytogenes*. The categories can be customized for normal and vulnerable consumers or depending on the customers' demands. If the growth potential of* L. monocytogenes* is not known, categories can be indicated based on product formulation or indicator bacteria.
- The risk of* L. monocytogenes* can be managed by setting suitable performance objectives consisting of maximum initial levels of* Listeria* combined with intended storage conditions and intended use. For products with high growth potential of* Listeria*, additional corrective actions may be needed. Some options suitable for large scale production are given in this guideline.

Links to legal documents, guides to good hygienic practices, and similar documents are given on the front page under “product-specific files.”

### 3.4. User-Friendliness and Relevance

All participants in the evaluation of the tool, both those within the team and external end-users, could open, log in to, and use the tool in their browser. The exercises worked well. Both the internal and external companies found the user-interface attractive. The prototype tool worked as intended and was found to be conceptually correct. From external companies the mean score was 4, with 5 as the theoretical max level. For user-friendliness the mean score was 3 from external companies and 3.64 in the project internal survey, which is considered good taking into account the fact that the restricted tool was a first version and that many of the respondents did not produce full meal salads. All respondents would recommend the tool to others, and most of the respondents assumed the tool would be useful for their own company if adapted to their processes and products.

### 3.5. Status and Further Development of the STARTEC Decision Support Tool

The STARTEC-tool was developed over a three-year period with the purpose of designing a prototype tool for the food industry, in particular small and medium size providers of complex ready-to-eat foods. Selected parameters within food safety, quality, nutrition, and costs for deli salads have been implemented in the tool so far, but the IT system is designed to implement any model at any step in the flow chart for any product. Other decision support tools have been on the market for decades, are more mature, and have different functions than the STARTEC prototype tool has. However, the STARTEC prototype tool illustrates how a multidisciplinary and intersectorial approach can provide a new framework and concept for tool development and indicate how such tools could be developed for the food industry in the future. At present, the tool is accessible with a password, free of charge. However, only few people have been given access so far, as the project is closed and the available resources for maintenance of the tool is limited after the project closed.

A main difference between the STARTEC-tool and other tools is the organization and the user-interface. Like other tools, the STARTEC-tool contain models and a database as well as a simulation module [26], but, in contrast to other tools, the user-interface of the STARTEC-tool starts with specific products and production processes rather than input parameters for models. The user-interface makes the STARTEC-tool product-specific and intuitive for the intended end-users both in terms of data input and in terms of simulation outputs. The product specificity makes it possible to insert relevant advice about corrective actions, easier to ensure that validated models and growth potentials are used for simulations and categorizations, and possible to assess the outputs in a multidisciplinary way based on product and company specific criteria.

On the other hand, the product specificity of the tool appears as a limitation as the tool needs to be filled with product-specific information to be used for another product. However, the database and simulation modules are designed to be general, which means that the information is valid for many products. For instance, prediction of food safety parameters with existing models based on pH, water activity, and so forth needs no other product adaptation than correct data for pH and water activity for the new product. For a company, the salary and energy costs will be the same for all products, which means that adaptation of the tool to other products within the company can be done with limited work. Product-specific models for quality scores, vitamin retention, and so forth require more adaptation to each product. However, the limiting factors in these cases are to obtain data and validated models for the specific products, rather than the possibility of including the data and models in the tool. Filter et al. [23] have identified ability and capacity for updating of knowledge databases as a challenge for use of IT expert systems for food safety in general and also suggested strategies for semiautomatic filling from community-driven food safety model systems. It can be foreseen that similar strategies can be applied in the future for nutrition and quality models. Thus, filling data is a limitation both for general and for specific tools.

The traffic light system of the STARTEC-tool provides information about compliance with specifications set for each category. According to feedback from users at conferences and industry [30, 49], this visualization in particular is considered useful for end-users, both because it gives an overview of the critical parameters and because it makes communication between the quality manager and other parts of the company easier. The feedback is in line with recent research on publications on the limiting factors for use of decision support tools. According to Filter et al. [23], recommendations are likely to improve the value of a decision support system. Koutsoumanis et al. [24] point out the importance of user-friendliness, in particular for users with limited knowledge in modelling. Further, Egan et al. [27] have reported that limited effect of HACCP and food hygiene training is that knowledge about food hygiene does not always lead to changes in attitudes and practices.

Some of the elements described above as characteristics for STARTEC are also considered in other tools. Tools like MicroHibro, FDA-iRisk, PMM-Lab, and ComBase have the option to build flow charts, at least sequences of steps where parameters like temperatures and pH can be changed in each step [26]. The option to simulate more than one parameter, for instance, growth of more pathogenic bacteria, was included in some tools already in their first versions. For instance, growth of different spoilage bacteria could be modelled in the first online version of the seafood spoilage predictor and combined models of* L. monocytogenes* and lactic acid bacteria were included some years later [17, 50]. Compliance with criteria was also included and presented as remaining shelf life before spoilage and predicted time until a log 2 cfu/g increase of* L. monocytogenes *was also included more than ten years ago. Similar functions are seen in other existing tools. Most tools focus on food safety parameters but, recently, combined models for pathogens and vitamin retention have been developed for the purpose of building multidisciplinary decision support tools based on real production and storage conditions [51]. Compared to existing tools, the STARTEC prototype tool has many of the same intentions as these tools, but it goes further in materializing the multidisciplinary aspects and product specificity as well as to take the industry view as a basis for design.

As mentioned above, the essential step for the validity of the STARTEC-tool is to categorize the simulation outputs correctly, which means to apply the right models, input parameters, and limit values for categorization. In the STARTEC prototype tool, a deterministic approach, which does not take variations into account, has been used for this purpose. Deli salads are complex matrixes where the ingredients have different growth potentials for various microbes [5, 13], which in turn can lead to underestimation of the growth and wrong categorization. This was taken into account in two ways in development of the STARTEC-tool. Our experimental data showed differences between parallel samples of* L. monocytogenes* counts of 0.5 log cfu/g or larger during storage [29], which indicate variations in the products in addition to measurement uncertainty. All data points were included in data fitting, indicating that the worst case scenarios were included in the models inserted in the tool. In addition, recommendations to use stricter criteria for categorization were included in the guidelines in cases where a higher safety margin was needed or the product was heterogeneous [31]. Another option would have been to include probabilistic models in the STARTEC-tool, as done in tools like GroPIN and Sym'previus [26]. The criteria for categorization would then need to be adapted to the probability of deviations due to variability as well. As a result, the complexity would increase, in particular if implemented for all food safety, quality, nutrition, and cost parameters. According to the feedback from test users that categorization and visualization of compliance with criteria are a strength of the STARTEC-tool, the more complex information from probabilistic models would still need to be visualized in a clear and intuitive way in the simulation outputs. A recent study of Guillier et al. [52] about the effects of advice for improved food safety and reduced food waste and energy consumption illustrates the relevance of probabilistic models for assessments of multidisciplinary questions on open and overall level and also how the simulation outputs can be visualized. The best tradeoffs between complexity, user-friendliness, and nuanced information for implementation in decision support tools for industry in the future will probably depend on which topics and end-users the tools address, technical options, and how the tools are designed.

Taken together, the STARTEC prototype tool developed is useful for demonstration of the concept and a good starting point for further development. The tool has been developed based on the end-users need, and elements of it may be useful to include in other tools as well. Therefore, the user-interface and approach applied in the STARTEC-tool can be of value also for other tools in order to improve their user-friendliness for food producers. The route for further development of the STARTEC-tool will depend on the interest from possible users and options for funding.

## 4. Conclusion

A prototype of an IT-tool has been successfully developed and validated in the project. Tools can be made specific and be suited for a few functions or generic and flexible so it can be adapted to many functions. In STARTEC, a generic and flexible structure was chosen which allows that more products, models, functions, and so forth can be included at a later stage. It is generally agreed that the STARTEC IT-tool prototype is promising with its multidisciplinarity, process/real food production approach, and flexibility to add more foods, models, and pop-up information. The prototype is useful for demonstration of the concept, but, on the other hand, it needs to be developed and/or filled with more validated models for more parameters to become really useful for the end-user. The strongest point of the prototype tool is that it is flexible and can be adapted to (a) the needs of any industry in terms of products, processes, and complexity of flow charts, (b) any multidisciplinary aspect and model for any parameter, and (c) different specifications and safety margins using customized categorization and the traffic light system. The tool is prepared to have a corrective action/support section, which can easily be developed more with new functionalities.

**Algorithm 1 (interview guide for mapping of decision processes).**

- How many versions of Lasagna do you produce? How much could the recipe be changed, without loss of quality/reputation/price/safety?
- Describe the product and process (dried/fresh pasta, kind of meat if any, are raw materials cut by a supplier or in the company, heat treatment in terms of time-temperature profile and storage conditions.) Are additives or preservation technologies used today? Is uneven distribution of additives or heat a challenge?
- Where do the raw materials for lasagna come from? Local/Imported materials? Are there challenges with some raw materials, in terms of availability, costs or quality/safety issues?
- How often does it happen that one of the raw materials needed is not available or too expensive to buy? How do you manage the situation – replace or take out the missing raw material, or not produce the product at all?
- Has the process been changed after the product development and upscaling? Do you see a need for changes in the process? If so, why?
- How is the quality and safety control of lasagna carried out on a day-to-day basis? Is every batch controlled? Which parameters are analyzed? Are all samples taken before the product is sent to the market, or is some sampling carried out in the supply chain or distribution chain as well?
- What is the shelf life of the product? Does it depend on time-temperature conditions during processing or storage, does it relate to raw material quality? Do you see a need for extended shelf life or improved quality/safety?
- What is the price of the product and raw materials? Do all consumers pay the same price? Could a higher price be obtained in case of a longer shelf life, better quality, etc? Is cost reduction a more realistic way to improve profit margin?
- Who are the customers and how do consumers treat the lasagna before they eat it? (Buy prewarmed or cold from the shop or central kitchen, microwave heating, eat it cold, etc.)
- If you get complaints, what is usually the reason? Could the deviation be foreseen if the quality and safety control had been broadened, or if an extra preservation step (or any other treatment) had been added? What is the rule; do the customers complain, or do they just stop to buy your product without saying anything?
- What are the views on food authority issues versus business related issues –what are the challenges
- How often and in which situations is decision support needed, where is support found today (in house/external advice).

## Figures and Tables

**Table 1:** Suggested border lines between red, yellow, and green categories for growth of *L. monocytogenes* in ready-to-eat foods fulfilling European Law, assuming initial contamination level 1 cfu/g.

Product category	Normal conditions	Extra safety margin for food intended served to vulnerable consumers	Comments
Green	<2.0 log cfu/g increase of Lm within shelf life	<0.5 log cfu/g increase of Lm within shelf life	Spoils before being unsafe
Yellow	2.0 log cfu/g increase of Lm by the end of shelf life	0.5 log cfu/g increase of Lm by the end of shelf life	Spoils and is unsafe at the same time
Red	>2.0 log cfu/g increase of Lm before the end of shelf life	>0.5 log cfu/g increase of Lm before the end of shelf life	Unsafe before being spoiled, with additional treatments needed to ensure safety
