# Corrigendum to “The STARTEC Decision Support Tool for Better Tradeoffs between Food Safety, Quality, Nutrition, and Costs in Production of Advanced Ready-to-Eat Foods”

**DOI:** 10.1155/2018/5189346

**Published:** 2018-09-26

**Authors:** Taran Skjerdal, Andras Gefferth, Miroslav Spajic, Edurne Gaston Estanga, Alessandra De Cesare, Silvia Vitali, Frederique Pasquali, Federica Bovo, Gerardo Manfreda, Rocco Mancusi, Marcello Trevisiani, Girum Tadesse Tessema, Tone Fagereng, Lena Haugland Moen, Lars Lyshaug, Anastasios Koidis, Gonzalo Delgado-Pando, Alexandros Ch. Stratakos, Marco Boeri, Cecilie From, Hyat Syed, Mirko Muccioli, Roberto Mulazzani, Catherine Halbert

**Affiliations:** ^1^Norwegian Veterinary Institute, Oslo, Norway; ^2^IRIS, Barcelona, Spain; ^3^University of Bologna, Bologna, Italy; ^4^Queens University, Belfast, UK; ^5^Matbørsen, Stokke, Norway; ^6^Kohinoor, Dublin 24, Ireland; ^7^Forno Romagnolo, Poggio Torriana, Italy; ^8^Halbert Research, Ireland

In the article titled “The STARTEC Decision Support Tool for Better Tradeoffs between Food Safety, Quality, Nutrition, and Costs in Production of Advanced Ready-to-Eat Foods” [1], the name of the fifth author was given incorrectly as Alessandra de Cecare. The author's name should have been written as Alessandra De Cesare. The revised authors' list is shown above.
